# Immunoprofiling of cytokines, chemokines, and growth factors in female patients with systemic lupus erythematosus– a pilot study

**DOI:** 10.1186/s12865-023-00551-6

**Published:** 2023-06-27

**Authors:** Laila A. Damiati, Iuliana Denetiu, Sami Bahlas, Samar Damiati, Peter Natesan Pushparaj

**Affiliations:** 1 Department of Biology, College of Science, University of Jeddah, Jeddah, Saudi Arabia; 2 Lab of Hematology, King Fahd Medical Research Centre, King Abdulaziz University, Jeddah, Saudi Arabia; 3 Department of Medicine, Faculty of Medicine, King Abdulaziz University, Jeddah, Saudi Arabia; 4 Department of Chemistry, College of Sciences, University of Sharjah, Sharjah, United Arab Emirates; 5 Center of Excellence in Genomic Medicine Research, Department of Medical Laboratory Technology, Faculty of Applied Medical Sciences, King Abdulaziz University, Jeddah, Saudi Arabia; 6 Centre for Transdisciplinary Research, Department of Pharmacology, Saveetha Dental College and Hospitals, Saveetha Institute of Medical and Technical Sciences, Chennai, India

**Keywords:** Systemic lupus erythematosus; cytokines, Chemokines, Growth factors, Luminex xMAP technology

## Abstract

Systemic Lupus Erythematosus (SLE) is a chronic autoimmune disease affecting different organ systems. This study aimed to determine the concentrations of 30 different human cytokines, chemokines, and growth factors in human plasma to understand the role of these markers in the pathogenicity of SLE using Luminex Multiple Analyte Profiling (xMAP) technology. Plasma samples were obtained from patients with SLE (n = 28), osteoarthritis (OA) (n = 9), and healthy individuals (n = 12) were obtained. High levels of TNF, IL-6, IFN-γ, INF-α, IL-4, IL-5, IL-13, IL-8, IP-10, MIG, MCP-1, MIP-1β, GM-CSF, G-CSF, EGF, VEGF, IL-12, IL-1RA, and IL-10 was detected in SLE patients compared with the OA and healthy control groups. xMAP analysis has been used to address the differential regulation of clinical heterogeneity and immunological phenotypes in SLE patients. In addition, complete disease phenotyping information along with cytokine immune profiles would be useful for developing personalized treatments for patients with SLE.

## Introduction

Systemic lupus erythematosus (SLE) is an autoimmune disease characterized by aberrant immune system activity, which causes different clinical symptoms such as heterogeneous renal, dermatological, and cardiovascular dysfunction [1], [2]. Genetic and environmental factors and cytokine imbalance are factors that trigger inflammation and induce organ damage [3]. In addition, a link between SLE and cytomegalovirus (CMV) has been suggested to affect disease development [4]. The SLE Disease Activity Index (SLEDAI) is one of the most commonly used disease activity measurements. It has less sensitive for changes, provides a high bar for improvement, and is based on symptom severity, regardless of the current severity in the patient [5]. The American College of Rheumatology (ACR) established the criteria for SLE classification. It contains laboratory biomarkers including anti-nuclear antibody (Ab) (ANA), anti-dsDNA Ab, complement (C2, C3, and C4), white blood cells, platelets, urinary casts, proteinuria, and hemolytic anemia [6]. The Systemic Lupus International Collaborating Clinics (SLICC) has validated an alternative system using a series of consensus exercises for symptomatology and laboratory results from real rheumatologic cases. The SLICC criteria require either meeting ≥ 4 of 17 criteria, including at least one clinical and one immunological criterion, or demonstrating biopsy for lupus nephritis proven with ANA or dsDNA [7], [8]. The SLICC classification criteria correlate with disease activity, capturing more manifestations that are also included in the SLEDAI [9]. Furthermore, although lupus is not a form of arthritis, it is one of the most common clinical features of SLE, which is usually non-erosive compared to rheumatoid arthritis (RA) [10]. Osteoarthritis (OA) is the most common type of noninflammatory arthritis. It is not rare for lupus patients to have OA, but significant conditions usually start at about age 50, which are unrelated to their lupus [11], [12].

Biomarkers play an important role in SLE diagnosis, classification, complications, and disease activity assessment. Therefore, finding an ideal biomarker for SLE is challenging, as it needs to meet different characteristics such as (i) reflecting the underlying pathophysiology, (ii) high predictive values, high sensitivity, and specificity, (iii) ability to monitor disease activity, (iv) reliability in tissues, cells, or fluid, and (v) stability and ease of detection [2], [13], [14].

Cytokines play a critical role in T cell proliferation, differentiation, activation, and function [15–17]. Secreted cytokines can be detected in the blood, saliva, urine, and target tissues, such as the skin and kidney. Most of these cytokines have pro-inflammatory properties; however, some have anti-inflammatory or immunomodulatory properties. However, as the pathogenesis of SLE is not well understood, it is not yet clear whether the levels of certain cytokines trigger the disease or are merely an epiphenomenon of multifunctional immune regulation, responses, cell death, or elimination of non-viable cell remnants [18]. In contrast, abnormal production of pro-inflammatory and anti-inflammatory cytokines has been reported as a potential therapeutic target for the treatment of SLE patients [11]. Cytokine profiling techniques provide a good way to evaluate the levels of various cytokines and assess their association with SLE disease progression and severity [19]. Therefore, finding an ideal biomarker for SLE is challenging, as it needs to meet different characteristics such as (i) reflecting the underlying pathophysiology, (ii) high predictive values, high sensitivity, and specificity, (iii) ability to monitor disease activity, (iv) reliability in tissues, cells, or fluid, and (v) stability and ease of detection [2], [13], [14].

Accordingly, this study aimed to determine the concentrations of 30 different human cytokines, chemokines, and growth factors in human plasma to understand the role of these markers in SLE pathogenicity using Luminex Multiple Analyte Profiling (xMAP) technology. Healthy controls and OA samples were used as comparators to gain insight into the nature of cytokine levels in SLE patients.

## Materials and methods

### Sample collection

Peripheral blood samples were obtained from healthy volunteers (n = 12), patients with knee osteoarthritis (OA) (n = 9), and patients with SLE (n = 28). The samples included in this study were from consecutive SLE patients who had no clinical evidence of OA, and the knee OA patients included in this study were examined clinically. The SLICC classification criteria [8] were determined at the last visit, and samples were collected. Disease activity was assessed using the SLEDAI, and a score of 3 or 4 was considered an active disease. Patient samples were collected from the Rheumatology Clinic of the King Abdulaziz University Hospital (KAUH), Jeddah, KSA, after obtaining written informed consent.

#### Sample processing

Plasma samples were obtained by centrifugation of the blood at 3,000 ×g for 10 min and stored immediately at -80 °C until further use.

### Multiple analyte profiling (xMAP)

Plasma samples isolated from normal controls, OA patients, and SLE patients were analyzed within 6 months for a variety of cytokines, chemokines, cytokine receptors, and growth factors using the Human Cytokine Magnetic 30-Plex Panel (Novex®) (Invitrogen, USA) according to the manufacturer’s instructions, as previously described (11). Briefly, magnetic beads coupled with antibodies for 30 different analytes were added and washed twice with 1X wash buffer. The standard was prepared by mixing the 16plex and 14plex solutions provided by the manufacturer, and a serial dilution protocol was used to prepare a range of standard solutions (1:3 serial dilution). Both standards and serum samples (1:2 dilution) were prepared and added to the washed beads in the designated wells of the Mylar plates and incubated in an orbital shaker at 500 rpm for 2 h. Between incubations with different antibodies, the plate was washed twice with the wash buffer. The plate was then incubated with secondary antibodies for 1 h and with streptavidin-RPE-coupled detection antibodies for 30 min. The plate was washed three times, resuspended in wash buffer, and analyzed using a MAGPIX® instrument (Luminex Corporation, USA).

### Statistical analysis

The raw data obtained for individual analytes were analyzed using the Luminex xPONENT® multiplex assay analysis software (Luminex Corporation, USA) to calculate the absolute concentration in the normal control, OA, and SLE groups. In addition, the concentration of each analyte was further analyzed using GraphPad Prism v.9 (GraphPad Software, USA) to compute the statistical significance using the Kruskal-Wallis test and Student’s t-test. Data was shown as mean ± SD and median (Interquartile range) for non-parametric data. All results are shown with 95%, 99%, and 99.9% accuracy for both the Kruskal–Wallis test and Student’s t-tests.

## Results

### Demographics, medications, and laboratory parameters

All the participants in this study were female (28 patients with SLE, 9 with OA, and 12 healthy controls). The median ages of patients with SLE, OA, and controls were 37 (with interquartile range 36–38), 50 (with interquartile range 49–51), and 43 (with interquartile range 42–44) years respectively. The clinical details for SLE were as follows: the mean duration of disease was 8,5 years; the disease activity was calculated using the SLE Disease Activity Index (SLEDAI), and the SLEDAI score to define active disease was 3 or 4. Fifteen patients were active, and 13 patients were inactive. Approximately 71% of SLE patients showed the presence of ANA and high levels of dsDNA. The patient characteristics, clinical details, and treatment regimens are shown in Table 1.

**Table 1:** Characteristics and clinical information of SLE patients

Parameters	Mean ± SD	Percentage (%)
Age (years)	37.3 ± 18	**-**
Disease duration (years)	8.5 ± 6.7	**-**
Disease activity (SLEDAI) (n) Active Inactive	5.2 ± 1.26 1.4 ± 0.51	- -
Complications (n) Diabetes Hypertension Cardiac diseases	3 12 6	11 43 21
Medications (n) Methotrexate Cellecept Prednisone	18 9 15	64 32 54
Immunological signs Presence of anti-nuclear Ab (ANA) (titer > 1:40 was considered positive) Abnormal anti-dsDNA Ab level (0-200 IU/ml) Low complement levels (C3) (0.75–1.65 g/L) Low complement levels (C4) (0.2–0.6 g/L)	20 501.7 ± 302 0.973 ± 0.19 0.2 ± 0.09	71 - - -
Complete blood count (CBC) White blood cell (WBC) (4.5–11.5 K/UL) Red blood cell (RBC) (4-5.4 M/µL) Hemoglobin (HB) (12–15 g/dL) Mean corpuscular volume (MCV) (80–94 fL) Mean corpuscular hemoglobin (MCH) (32–36 pg) Platelet (PLT) (150–450 K/UL)	5.96 ± 1.9 4.44 ± 0.79 12.42 ± 5.31 79.05 ± 10.09 25.96 ± 4.11 260.17 ± 73.31	-
Kidney and liver function tests Urea (3.2–8.2 mmol/L) Creatinine (53–115 µmol/L) Alanine aminotransferase (ALT) (12–78 U/L) Aspartate aminotransferase (AST) (15–37 U/L)	7.23 ± 5.25 83.11 ± 44.33 22 ± 9.61 18.34 ± 5.54	-

### Th cytokine profiles

**Fig. 1:**
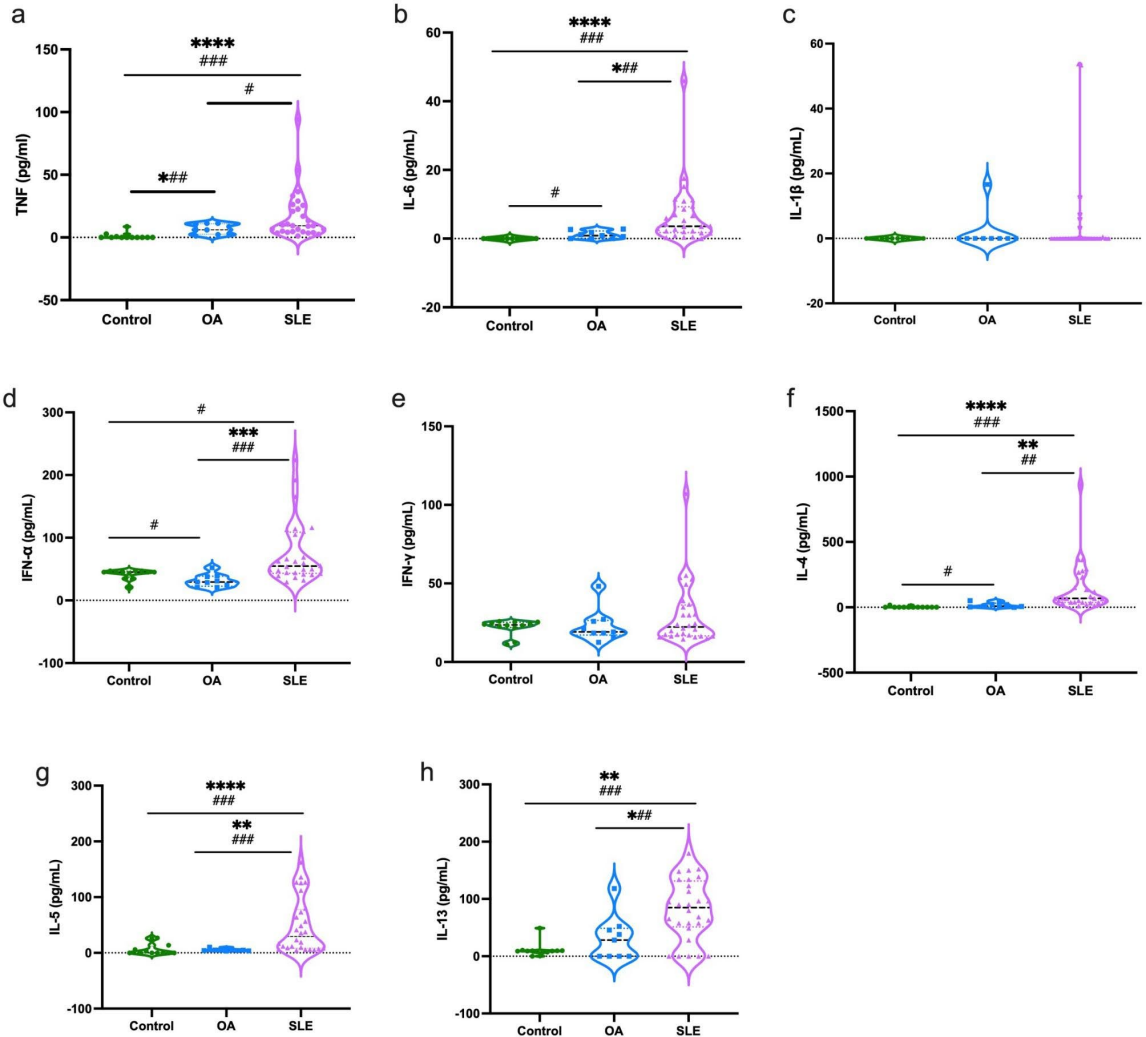
Levels of Th1 (TNF, IL-6, IL-1β, IFN-α, IFN-γ), and Th2 (IL-4, IL-5, IL-13) cytokines in the plasma of control, OA, and SLE groups. Graph shows the median by Kruskal Wallis test, where *= P < 0.05, **=P < 0.01, ***=P < 0.001, ****=P < 0.0001 0001 and Student’s t-test #=P < 0.05, ##=P < 0.01, ###=P < 0.001

Plasma levels of Th1 (TNF, IL-6, IL-1β, IFN-α, and IFN-γ) and Th2 (IL-4, IL-5, and IL-13) cytokines in the SLE, OA, and healthy control groups were quantified using a magnetic 30-plex panel based on Luminex xMAP technology. Here, we observed high expression levels of TNF (a), IL-6 (b), IFN-α (d), IL-4 (f), IL-5 (g), and IL-13 (h) in SLE patients compared to controls (healthy and OA). No expression of IL-6, IK-1β, or IL-4 was observed in the control group. However, there was no significant difference in the expression of IL-1β (c) and IFN-γ (e) (Th1) between all groups. In addition, the comparison between the groups showed that there was a significant difference between the OA and SLE groups in the levels of TNF- and IFN-α and between the control and OA groups in the levels of IFN-α and IL-4 (Fig. 1).

### Chemokine profiles

**Fig. 2:**
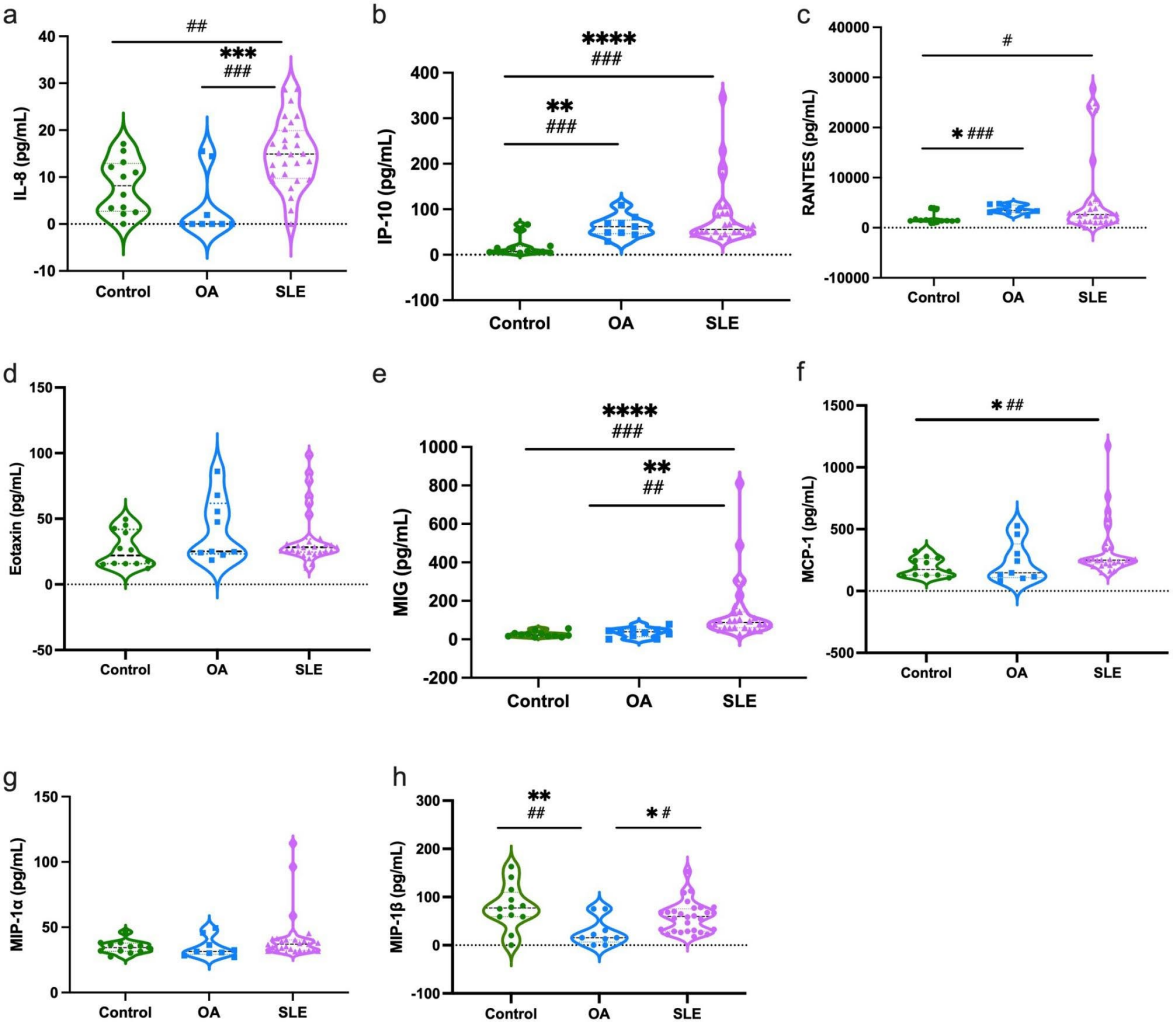
Levels of chemokines (IL-8, IP-10, RANTES, Eotaxin, MIG, MCP-1, MIP-1α, MIP-1β) in the plasma of control, OA, and SLE groups. Graph shows the median by Kruskal Wallis test, where *= P < 0.05, **=P < 0.01, ***=P < 0.001, ****=P < 0.0001 0001 and Student’s t-test #=P < 0.05, ##=P < 0.01, ###=P < 0.001

Similar to the cytokines, some chemokines were highly expressed in patients with SLE compared to the control or OA groups, such as IL-8 (a), IP-10 (b), MIG (e), MCP-1 (f), and MIP-1α (h). However, RANTES (c) increased in OA compared to healthy controls. The MIP-1β (h) levels were significantly higher in the control and SLE groups than in the OA group. No significant difference was observed in the expression of Eotaxin (d) or MIP-1α (g) between the groups. In addition, a comparison between groups showed that there was a significant difference in the expression of IL-8 and RANTES between the control and SLE groups (Fig. 2).

### Growth factors profiles

**Fig. 3:**
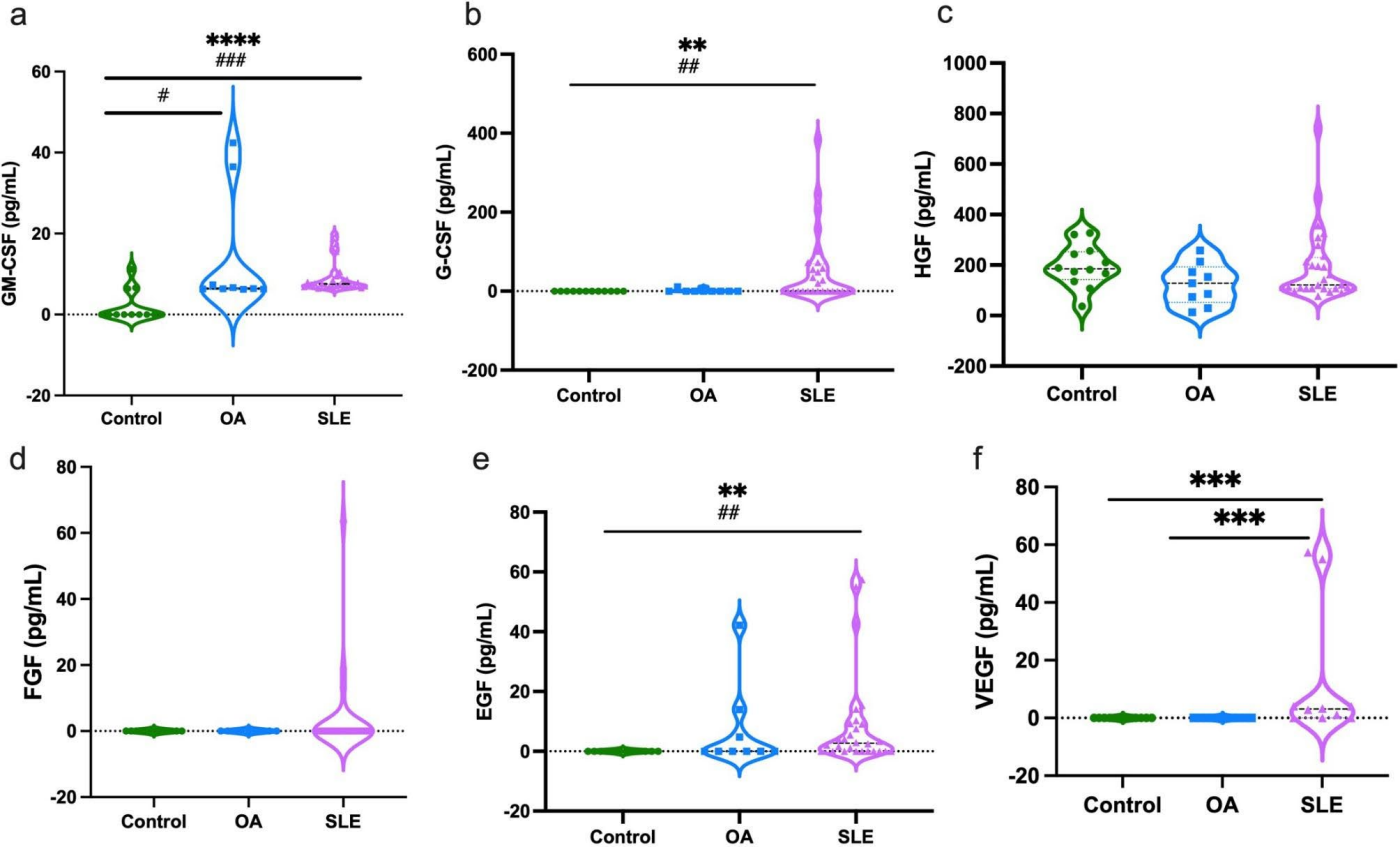
Levels of growth factors (GM-CSF, G-CSF, HGF, FGF, EGF, VEGF) in the plasma of control, OA, and SLE groups. Graph shows the median by Kruskal Wallis test, where *= P < 0.05, **=P < 0.01, ***=P < 0.001, ****=P < 0.0001 and Student’s t-test #=P < 0.05, ##=P < 0.01, ###=P < 0.001

Among the growth factor measurements, GM-CSF (a), G-CSF (b), EGF (e), and VEGF (f) levels were significantly higher in the SLE group than in the controls. HGF (c) and FGF (d) levels did not differ between the groups. In addition, the comparison between the groups showed that there was a significant difference in the levels of GM-CSF between the control and OA groups. G-CSF, EGF, VEGF, and FGF markers were not detected in control and some OA patients (Fig. 3).

### Anti-inflammatory and other pro-inflammatory cytokine profiles

**Fig. 4:**
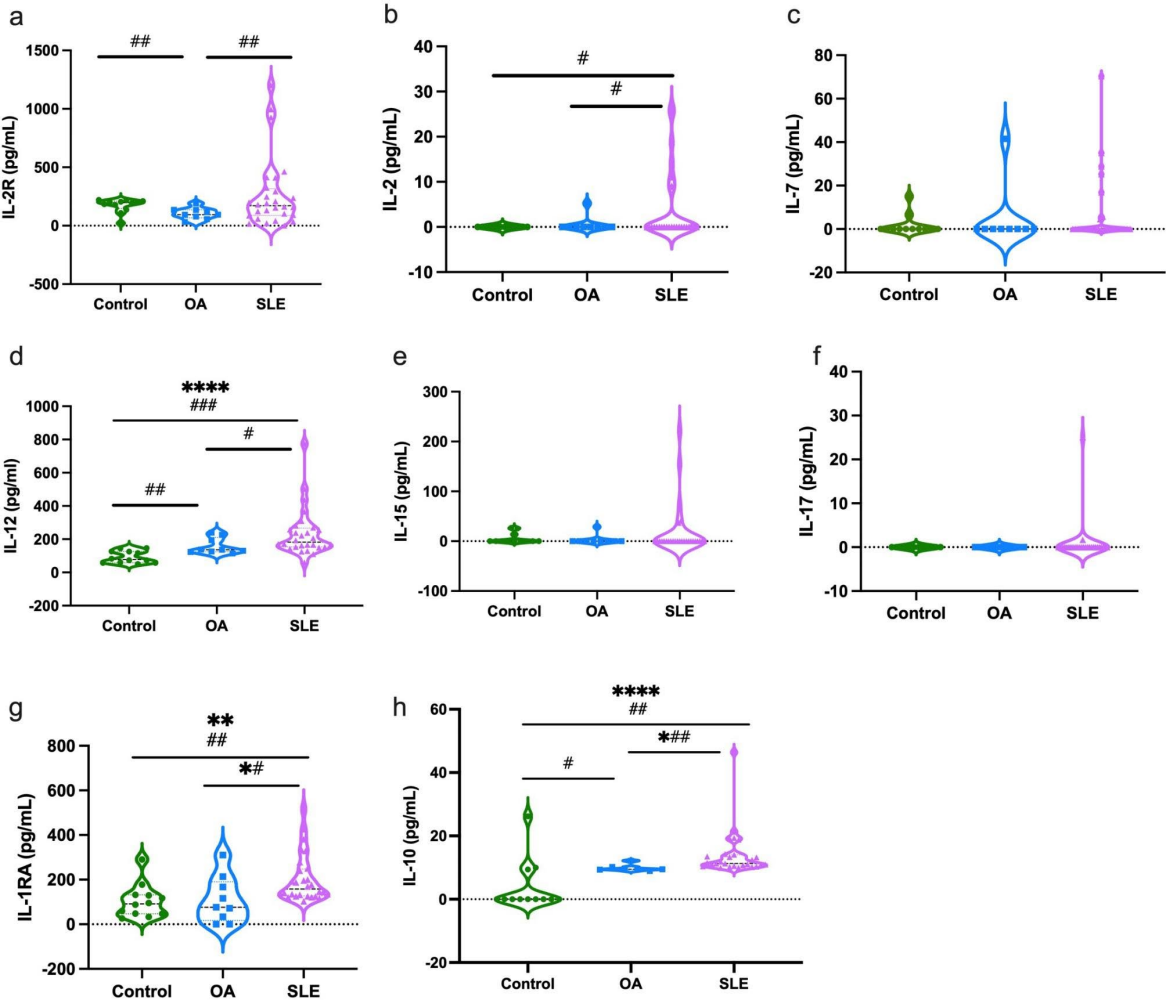
Levels of pro-inflammatory (IL-2R, IL-2, IL-7, IL-15, IL-17) and anti-inflammatory (IL-1RA, IL-10) mediators in the plasma of control, OA, and SLE groups. Graph shows the median by Kruskal Wallis test, where *= P < 0.05, **=P < 0.01, ***=P < 0.001, ****=P < 0.0001 and Student’s t-test #=P < 0.05, ##=P < 0.01, ###=P < 0.001

IL-12 (d) was highly expressed in SLE patients compared to the control groups, unlike IL-2R (a), IL-2 (b), IL-7 (c), IL-15 (e), and IL-17 (f). The IL-1RA (g) and IL-10 (h) mediators were higher in the SLE group than in the OA and control groups. IL-2, IL-15, IL-17 and IL-7 were not detected in control and some OA or SLE patients. In addition, the comparison between the groups showed a significant difference between the control and SLE groups in the levels of IL -2 and between the control and OA groups in the levels of IL -2R, IL -12, IL -10, and the levels of IL -2 and IL -12 between the OA and SLE groups (Fig. 4).

## Discussion

The abnormal activity of cytokines plays an important role in autoimmune disease (e.g., SLE) pathogenicity, and multiplex assays help to assess the abnormal biological activity of cytokines, chemokines, and growth factor levels [19–22]. Activation of the major components of the innate and adaptive immune systems has been observed in patients with SLE. Increased levels of various cytokines are also observed during the disease course [12]. In the current study, an array of cytokines, chemokines, and growth factor levels were evaluated using the Human Cytokine Magnetic 30-Plex Panel in 28 SLE patients, 9 with OA, and 12 healthy controls. OA or arthralgia occurs in 95% of patients with SLE at some point during the course of the disease [19], [22]. Therefore, in this study, we compared SLE and OA patients to identify common markers that may play a role in the development of OA in SLE patients. Patients with SLE had significantly increased levels of Th1 and Th2 associated cytokines including TNF, IL-6, IFN-γ, IL-4, IL-5, and IL-13. A comparison between the groups also showed significant differences in the levels of TNF and IFN-α. TNF can either promote or modulate autoimmunity; however, it has been shown to be upregulated in SLE patients compared to healthy controls and is correlated with disease activity [23–25]. A previous study by Xiang et al. showed high expression level of IL-1β, IL-18, IL-6, IFN-α, IL-10, and TNF compared to healthy controls. In the same study, they found that TGF-β1 levels in SLE patients were significantly lower than those in controls [26]. Another study by Hashemi et al. found that the serum levels of TNF-α, IL-2, IL-4, and IL-5 were significantly decreased in SLE patients who underwent an 8-weeks exercise training program compared to those in the control group. In addition, the levels of IL-10, IL-13, and IL-22 were significantly higher in the control group than in the intervention group after 8-weeks of exercises [27]. Although OA is a noninflammatory form of arthritis, we found that pro-inflammatory cytokines TNF-α and IL -6 were significantly higher in OA compared with healthy controls. Molnar et al. reviewed in detail the cytokines and chemokines involved in the pathogenesis of OA and highlighted the studies showing the systemic increase in pro-inflammatory cytokines and chemokines in OA patients compared to healthy controls [28].

Chemokines are a group of cytokines with low molecular weights that direct the chemotaxis of target cells. They play a role in the pathogenesis of SLE and related complications, especially lupus nephritis [29]. The concentrations of IL-8, IP-10, MIG, and MCP-1 were significantly higher is SLE patients compering than in the OA and control groups. On the other hand, here, the level of RANTES was higher in OA group compering to SLE group However, comparisons within groups showed that there was a significant difference between the control and SLE groups in IL-8 and RANTES. Similar to Yoshio et al., the mean concentrations of IL-6, IL-8, IP-10, MCP-1, G-CSF, and GM-CSF were higher in central neuropsychiatric SLE than in the sera, and the level of RANTES was much lower than that in the control [30]. Eotaxin and MIP-1α levels were not significantly different between groups. However, a study by Novikov et al. showed that the expression of otaxin and MIP-1α was significantly lower in patients with SLE than in those with rheumatoid arthritis (RA) group [31].

For growth factors expression, the GM-CSF and G-CSF are the key survival factors for granulocytes [32]. They have been shown to inhibit neutrophil apoptosis in healthy subjects in vitro at various concentrations [33]. GM-CSF deficiency in mice leads to the development of an SLE-like disease that correlates with impaired apoptotic cell phagocytosis [34]. EGF has been suggested for monitoring kidney injury in the setting of demonstrations of SLE [35]. Previous studies have shown that high VEGF levels may correlate with disease activity in SLE and lupus nephritis. However, high urinary VEGF expression may represent the ongoing process of fibrosis and tissue repair in lupus nephritis kidneys [36], [37]. A study by Adhya et al. showed that VEGF levels could be a useful marker to detect disease activity in the urine of lupus nephritis patients compared to non-renal SLE patients [38]. VEGF, TNF-α, HGF, and FGF are potent mitogens with angiogenic activity [39], [40]. Hrycek et al. showed that FGF levels were low in patients with newly diagnosed untreated SLE [39]. In the present study, GM-CSF, G-CSF, EGF, and VEGF levels were significantly higher in the SLE group than in the OA and healthy control groups. There was a significant difference in the levels of GM-CSF. However, HGF and FGF levels did not differ between the groups.

IL-12 is a pro-inflammatory cytokine that induces Th cell differentiation into Th1 cells [41]. It also induces different cytotoxicity pathways and the expression of cytotoxic mediators by amplifying the production of IFN-γ [42]. High levels of IL-12 have been observed in patients with SLE compared with controls [43]. IL-2 is a proinflammatory mediator that plays an important role in immune cell activation and peripheral tolerance. It has been suggested to exhibit therapeutic efficacy in patients [44], [45]. IL-15 plays a role in B cell antibody production, which is increased in the serum of SLE patients [46]. Moreover, IL-2R is correlated with other markers that are potential targets of IL-15 [47]. IL-7 plays an important role in T-cell development, homeostasis, and immune tolerance, suggesting the use of the IL-7/IL-7Rα pathway as a target for autoimmune disease treatment [48]. IL-17 and Th17 cells are involved in SLE pathogenesis [49]. However, previous studies have shown no increase in IL-17 expression and no correlation with disease activity [50], [51]. In the current study, IL-12 was the only pro-inflammatory mediator that showed high expression in SLE patients compared to that in healthy controls. On the other hand, IL-2R, IL-2, IL-7, IL-15, and IL-17 mediators did not show any significant differences compared with the control group. IL-1RA and IL-10 mediators were higher in the SLE group than in the OA and control groups. In addition, the comparison between the control and OA groups showed significant differences in the values of IL-2R, IL-2, IL-12, and IL-10, and in the values of IL-2R, IL-2, and IL-12 between OA and SLE, as well as in the values of IL-2 between the control and OA groups. Similarly, Toit et al. showed high expression of IL -1RA in SLE patients with myocardial damage [52], and IL -10 level were higher in SLE patients than in controls [16].

## Conclusions

In conclusion, the current study demonstrates that SLE is a complex disease, and the underlying disease mechanism remines unclear. Further analysis is required to understand whether there is any relationship between the high expression of some markers in SLE and OA patients, but not in controls. This indicates that patients with SLE may be able to develop OA at some stage during their disease course. A better understanding of these biological factors and their expression may provide essential clues to the pathogenic pathways and open new avenues for more effective therapeutics. However, the small sample size in the present study may be considered a bias, and further studies are needed to understand the cytokine network and patterns both ex vivo in human SLE samples and in vivo in animal models. Additionally, more information on disease phenotyping would be helpful in planning personalized treatment.

## Data Availability

The datasets used and/or analyzed during the current study available from the corresponding author on reasonable request.

## Abbreviations

IL-1RA: Interleukin 1-receptor antagonist
IL-2R: Interleukin-2 receptor
ACR: American College of Rheumatology
ALT: Alanine aminotransferase
ANA: Anti-nuclear antibody
AST: Aspartate aminotransferase
CBC: Complete blood count
CCL11: C-C motif chemokine Ligand 11 or Eotaxin
CMV: Cytomegalovirus
EGF: Epidermal growth factor
FGF: Fibroblast growth factor
G-CSF: Granulocyte-colony stimulating factor
GM-CSF: Granulocyte Macrophage -Colony Stimulating factor
HB: Hemoglobin
HGF: Hepatocyte growth factor
IFN-α: Interferon alpha
IFN-γ: Interferon gamma
IL-1β: Interleukin-1 beta
IL: Interleukin
IP-10: Interferon γ-inducible protein-10
MCH: Mean corpuscular hemoglobin
MCP-1: Monocyte chemotactic protein-1
MCV: Mean corpuscular volume
MIG: Monokine induced by gamma interferon
MIP-1α: Macrophage inflammatory protein alpha
MIP-1β: Macrophage inflammatory protein-1 beta
OA: Osteoarthritis
PLT: Platelet
RA: Rheumatoid arthritis
RANTES: regulated on activation, normal T cell expressed and secreted
RBC: Red blood cell
SLE: Systemic Lupus Erythematosus
SLEDAI: SLE Disease Activity Index
SLICC: Systemic Lupus International Collaborating Clinics
TNF-α: Tumor necrosis factor alpha
VEGF: Vascular endothelial growth factor
xMAP: Luminex Multiple Analyte Profiling
